# Direct Conversion of Fibroblast into Neurons for Alzheimer’s Disease Research: A Systematic Review

**DOI:** 10.3233/JAD-230119

**Published:** 2023-09-26

**Authors:** Roman Sattarov, Håkan Toresson, Camilla Orbjörn, Niklas Mattsson-Carlgren

**Affiliations:** a Department of Clinical Sciences Malmö, Clinical Memory Research Unit, Lund University, Lund, Sweden; b Department of Neurology, Skåne University Hospital, Lund University, Lund, Sweden; c Wallenberg Center for Molecular Medicine, Lund University, Lund, Sweden

**Keywords:** Adult human dermal fibroblasts, Alzheimer’s disease, amyloid-β, *APOE*, *APP*, familial Alzheimer’s disease, human induced-neurons, sporadic Alzheimer’s disease, tau

## Abstract

**Background:**

Alzheimer’s disease (AD) is a prevalent neurodegenerative disorder without a cure. Innovative disease models, such as induced neurons (iNs), could enhance our understanding of AD mechanisms and accelerate treatment development. However, a review of AD human iN studies is necessary to consolidate knowledge.

**Objective:**

The objective of this review is to examine the current body of literature on AD human iN cells and provide an overview of the findings to date.

**Methods:**

We searched two databases for relevant studies published between 2010 and 2023, identifying nine studies meeting our criteria.

**Results:**

Reviewed studies indicate the feasibility of generating iNs directly from AD patients’ fibroblasts using chemical induction or viral vectors. These cells express mature neuronal markers, including MAP-2, NeuN, synapsin, and tau. However, most studies were limited in sample size and primarily focused on autosomal dominant familial AD (FAD) rather than the more common sporadic forms of AD. Several studies indicated that iNs derived from FAD fibroblasts exhibited abnormal amyloid-β metabolism, a characteristic feature of AD in humans. Additionally, elevated levels of hyperphosphorylated tau, another hallmark of AD, were reported in some studies.

**Conclusion:**

Although only a limited number of small-scale studies are currently available, AD patient-derived iNs hold promise as a valuable model for investigating AD pathogenesis. Future research should aim to conduct larger studies, particularly focusing on sporadic AD cases, to enhance the clinical relevance of the findings for the broader AD patient population. Moreover, these cells can be utilized in screening potential novel treatments for AD.

## INTRODUCTION

Alzheimer’s disease (AD) is the most common neurodegenerative disorder, with a rapidly increasing prevalence, mainly due to increased life expectancy [1]. Neuropathologically, AD is characterized by the accumulation of amyloid-β (Aβ) in plaques and tau in neurofibrillary tangles in the brain. These changes are associated with neuronal loss and cognitive impairment [2]. Aβ accumulation is established before cognitive impairment [3], while aggregation of neurofibrillary tangles and loss of neurons and synapses appear to accrue later, in parallel with progression of cognitive decline [4–6]. When symptoms start before age 65, the syndrome is called early-onset AD (EOAD), which represents ∼5% of AD cases. In a small fraction of patients (<1%), who often have EOAD, the disease is caused by autosomal dominant mutations in the amyloid precursor protein (*APP*), presenilin 1 (*PSEN1*), or presenilin 2 (*PSEN2*) genes, causing familial forms of AD (FAD) [7]. Most patients (>99%) have sporadic AD (SAD) which typically occurs past the age of 65, often referred to as late-onset AD (LOAD) [8]. A combination of risk factors, including age, genetics, and environmental factors contribute to SAD [9]. The strongest genetic factor is the *ɛ*4 allele of the apolipoprotein E (*APOE*) gene. The pathobiological function of the *ɛ*4 allele is complex, but it may affect the clearance pathway of Aβ, as well as potentially also affect tau-mediated neurodegeneration and neuroinflammation [10]. The risk for AD increases and the age of onset of AD declines with an increased number of *ɛ*4 alleles.

There is no cure for AD. Several phase III studies of putative disease-modifying treatments have failed [11–13], but a few recent studies have demonstrated potentially beneficial effects of anti-Aβ immunotherapies [14–16]. Innovative disease models may accelerate the development of efficient therapies, by providing new knowledge about disease mechanisms and inform on candidate treatments. The field has come a long way since the first transgenic animal models for AD were established. Now there are multiple models tailored for specific research questions, attempting to reproduce biochemical and histological changes at the cellular level or to model cognitive impairment, or the temporal and spatial course of neurodegeneration [17, 18]. There are several rodent models of early-stage AD with FAD-associated mutations. Most mice models are transgenic mice that express human *APP* alone or in combination with human *PSEN1* (resulting in the formation of Aβ plaques), or human *MAPT* (resulting in the formation of neurofibrillary tangles) [19, 20]. Even though rodent models have been important for AD research, the translation rate of candidate drugs from a promising finding in animal models to an approved drug in humans is very low. So far, only two disease-modifying AD treatments have been approved by the US food and drug administration (FDA) (Aducanumab and Lecanemab) [21, 22]. There are several possible reasons for why findings from animal models may not always translate to humans [23]. One challenging aspect is that rodents are significantly different from humans in physiology and molecular biology. For example, genetic differences between humans and rodents may have significant effects, since binding sites of RNA-binding proteins associated with neurodegeneration are not well conserved and RNA-processing alterations are not fully recapitulated in rodent models [23]. Differences in lifespans between humans and rodents might also contribute to why animal models fail to capture key aspects of the pathology of late-onset diseases such as AD [24]. However, rodent models overexpressing mutant *APP* may exhibit learning and memory deficiencies, as well as altered behavior, such as decreased exploration [17], which could be related to the accumulation of Aβ plaques in specific brain regions, such as the hippocampus and cortex [18]. Not all transgenic rodent models display the same behavioral alterations, and differences may depend on the specific *APP* mutation, the level of *APP* expression, the age of the animals or other factors [25]. In sum, rodent models provide valuable insights into the pathophysiology of AD, but they do not perfectly replicate the human disease and need to be combined with other scalable models.

### Induced pluripotent stem cell-derived neurons

**Fig. 1:**
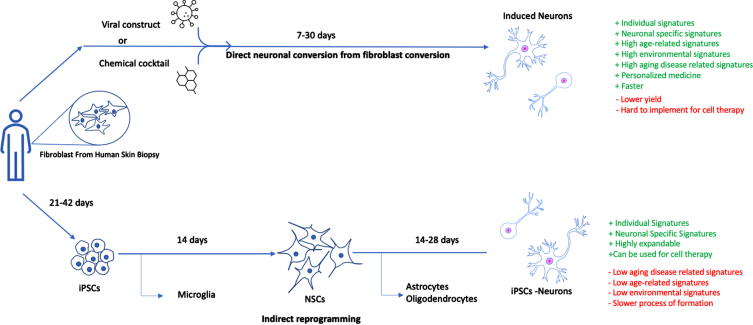
Comparison between direct neuronal conversion and indirect reprogramming. Comparison of direct and indirect reprogramming to the neuronal cell-fate. Somatic cells from patients can be directly or indirectly reprogrammed to iNs. Direct reprogramming can be achieved by overexpression of transcription factors (such as Brn2, Ascl1, and Myt1) or other supplemental neurogenesis factors, which reprogram fibroblast cells under 30 days. In contrast, somatic cells can be reprogrammed to induced pluripotent stem cells (iPSCs) with Yamanaka transcription factors [101]. Such methods lengthen the reprogramming timeline as quality control assays for karyotypic stability and pluripotency must be conducted. Advantages and limitations of each method are summarized in the figure.

Human induced pluripotent stem cell-derived neurons (hiPSC-Ns), induced neural stem cells (iNSCs), and induced neurons (iNs) provide human cellular models to complement rodent models of AD. Postmortem tissues, e.g., skin, or other biopsy sources, have been used to generate iPSCs and iNs for studies of genetic effects on neurotoxicity [26, 27] (Fig. 1). With well-defined factors for cell reprogramming, fibroblasts can be reprogrammed to iPSCs by ectopic expression of four embryonic transcription factors (TFs) (Oct4, Sox2, Klf4, and c-Myc), which revert the cells back to an embryonic-like state [28]. Patient-derived neurons generated using iPSCs are interesting as disease models [29], and have been used both for LOAD and FAD models [30], e.g., to investigate protein aggregate toxicity, and amyloid-β protein precursor (AβPP) processing [29]. Phenotype studies have shown that iPSC-derived forebrain neurons from AD patients with *APP* and *PSEN1* mutations have a higher Aβ_42_/Aβ_40_ ratio [31] (reflecting a relative increase in Aβ_42_, which is generally considered to be a more pathogenic Aβ peptide variant), intracellular accumulation of Aβ oligomers [32], increased levels of Aβ peptides, increased levels of total tau (t-tau) and hyperphosphorylated tau (p-tau) proteins [33], increased numbers of large and very large early endosomes [33], and increased reactive oxygen species production [34]. iPSCs have even been suggested to have a potential for replacement cell therapies for the central nervous system (CNS) [35, 36]. Pilot experiments have shown promising results for iPSC transplants in Parkinson’s disease, and in animal AD models, using human iPSC-derived astrocytes and neurons [37–39]. There is also hope that iPSCs may be used for personalized medicine, with identification and treatment of patient-specific factors [36]. However, for modelling (rather than treatment) of age-related diseases such as AD, it is concerning that reprogramming into pluripotent cells results in loss of age- and environment-dependent cellular and epigenetic signatures, producing young neurons with reset cellular age [33, 40].

### Induced neurons

For the modelling of age-associated neurodegenerative diseases, an alternative approach exists, through a process called direct conversion (Fig. 1). The first directly converted neurons were created by Vierbuchen and colleagues in 2010 from mouse fibroblasts, using what is now referred to as the BAM reprogramming factor, which includes the TFs Brn2, Ascl1, and Myt1 L. Shortly thereafter, these BAM factors were used to convert human fibroblast into neurons [41]. Since the initial work in 2010, iNs have been generated through a number of strategies involving the use of various combinations of miRNAs and small molecule cocktails. The main advantage of direct conversion rather than iPSCs is that the direct conversion of one somatic cell type into another skips the proliferative stem cell intermediate. This makes the production process faster and easier and also allows the resulting iNs, unlike iPSCs, to maintain the donor’s ageing signature [42, 43]. Ageing is associated with different epigenetic changes, with alterations in chromatin states triggered by changes in DNA methylation, post-translational modifications of histones, and histone protein levels [44]. The preservation of such changes makes iNs more desirable for modelling of age-related diseases, which may be influenced by epigenetic factors. For example, epigenetics may contribute to the heterogeneous clinical presentation of AD in patients with similar genetic backgrounds [45]. Although initial twin studies of global DNA methylation in brain tissue (from the temporal cortex, hippocampus, and frontal cortex) of patients with AD were inconclusive [46], it has been suggested that DNA methylation in AD can be cell-type specific, with methylation changes in neurons, glial cells and astrocytes, but not in interneurons or microglia [47]. Another study also demonstrated that DNA methylation alterations in AD are dependent upon the particular cell type [45]. Furthermore, several studies have shown common DNA methylation alterations in specific genes, such as *ANK1*, *BIN1*, *RPL13*, *RHBDF2*, and *CDH23* in AD [48–50].

Additionally, histone modifications have effects on psychiatric and neurological conditions, including AD [51]. For example, histone modifications may contribute to elevated expression of *RELN* gene, which is involved in synaptic plasticity and memory and has been implicated in AD [52]. The possibility to preserve these epigenetic changes makes a strong case for iNs over iPSCs for modelling neuronal pathology in LOAD in particular (where ageing-related factors are likely to be more important than in autosomal dominant AD) [53]. INs for AD have therefore been discussed as a clinically relevant disease model [54].

### Aims

Different iN model systems have been described over the last few years in AD studies, but there is a lack of a systematic review of the literature in this area, summarizing both the methods used and the results with relevance for AD. The purpose of this study was therefore to perform a systematic review of direct reprogrammed neurons as a cell model for AD. We aimed to clarify the number and scope of published studies, describe published methods to reprogram cells for iNs in AD and give a comprehensive description of published results, in terms of the converted cells’ characteristics, the onset of differentiation, the phenotypes tested to mimic typical AD features, and alternative models used to validate the findings. We also discuss the potential limitations of the use of the iNs and identify key knowledge gaps for future studies.

## METHODS

### Data source

**Fig. 2:**
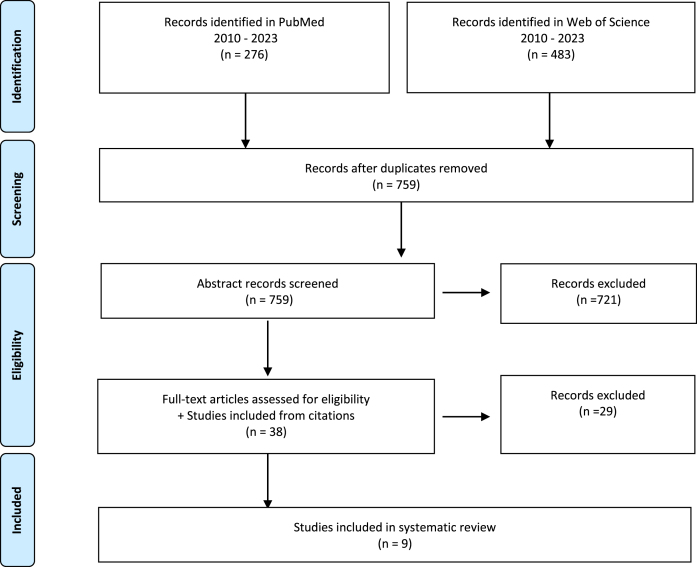
Screening Flow Diagram. For records identification in PubMed the following search criteria were used: (“alzheimer’s”[Title/Abstract] OR “alzheimer”) AND (((“induced neurons”[Title/Abstract]) OR “induced neuronal cell” OR “induced neuron” OR (“reprogrammed”[Title/Abstract]))). For records identification in Web of Science the following search criteria were used: #1 AND #2 AND #3 AND #4. Where #1 ((TI = (alzheimer’s)) OR AB = (alzheimer’s)), #2 ((TI = (alzheimer)) OR AB = (alzheimer)), #3 (((((TI = (induced neurons)) OR TI = (induced neurons))) OR TI = (induced neuronal cell)) OR TI = (induced neuron)) OR TI = (reprogrammed), #4(((((AB = (induced neurons)) OR AB = (induced neurons))) OR AB = (induced neuronal cell)) OR AB = (induced neuron)) OR AB = (reprogrammed). Inclusion and exclusion criteria are presented in the main text.

The search for relevant articles was conducted on April 7, 2023, using two major databases, PubMed and Web of Science, to identify relevant articles on iNs in AD (Fig. 2). Our search terms were [“Alzheimer’s” OR “Alzheimer”] AND [“induced neurons” OR “induced neuronal cell” OR “induced neuron” OR “reprogrammed”] which we searched for in the title or abstract of articles. We retrieved 276 and 483 articles from PubMed and Web of Science, respectively. We manually screened the studies from each database based on their titles and abstracts and removed any duplicates. Two authors then assessed the full-text articles for eligibility based on the inclusion and exclusion criteria described below. We followed the Preferred Reporting Items for Systematic Reviews and Meta-Analyses (PRISM) 2020 guidelines checklist (see Supplementary Table 1) recommended for planning and conducting systematic reviews.

### Inclusion/exclusion criteria

Inclusion criteria were established to ensure that studies were relevant to the research question. Only studies published in English from January 2010 to April 2023 were included (the starting date was set due to the pioneering work of Vierbuchen on the neuronal reprogramming method [55]). To be eligible, studies needed to meet the following criteria: 1) use direct conversion methods, as one of the methods, 2) be *in vitro* studies (experiments on cells, not on tissue), 3) use human cell lines, and 4) involve confirmed AD. Exclusion criteria were also established to ensure that the studies included in the analysis were of high quality and relevance. Studies were excluded if they: 1) were purely animal studies, 2) were review articles, 3) did not involve direct reprogramming, or used an intermediate step converting fibroblasts to iPSCs and then used direct methods on these cells. Despite a comprehensive search, only a limited number of studies met our inclusion criteria. We conducted a detailed analysis of these studies, in terms of their methods, results and limitations.

### Synthesis methods

The PRISMA statement is a widely recognized and endorsed framework for reporting systematic reviews. In our study, we followed the PRISMA guidelines to ensure the transparent reporting of our review methods, results, and conclusions. We used a comprehensive search strategy, documented our inclusion and exclusion criteria, and detailed our data extraction and synthesis procedures. We also evaluated the risk of bias for each included study and summarized the findings. However, due to the limited number of studies, we were unable to perform subgroup analyses or sensitivity analyses to statistically assess heterogeneity of the study results.

### Bias assessment

The reviewers who reviewed the identified papers assessed the individual risk of study bias by interpretation of the study methods, as described in the papers. We also considered the hypothetical risk of bias due to missing studies or missing results within studies, following the PRISMA guidelines [56].

### Certainty assessments

For certainty assessment, we focused on study limitations, consistency of effects and publications bias. These factors were assessed across the identified studies by the reviewers.

## RESULTS

### Overview of the included studies

Nine studies [35, 57–64] met the criteria to be included in this review. The key features of the studies are summarized in Table 1. All studies used human fibroblasts from skin biopsies, either post- or ante-mortem, as the starting material for conversion to iNs (see Table 2). Two research teams were represented with several papers (one team with three papers: Traxler et al. [62], Mertens et al. [64], and Herdy et al. [63]; and another team with two papers: Kim et al. [60] and Kim et al. [61]). In their respective papers, these teams used overlapping methods and overlapping source material for iNs. Details of the different approaches for iNs conversion are summarized in Table 3. All studies demonstrated to a varying degree that they obtained mature neurons. In all cases, the reported iNs were positive for β-III tubulin, NeuN, and MAP-2, or β-III tubulin and NeuN, with some studies also reporting other neuronal markers (Table 1). All studies except [57] provided immunofluorescence images with mature neuronal markers, which showed that the iNs exhibited round or pyramidal somas, condensed nuclei, long axons, and multiple neurites. Below we summarize key components of the study design, starting with the most recent publications. In summary, Kim et al. [61], Traxler et al. [62], Herdy et al. [63], and Mertens et al. [64] used a small molecule-enhanced vector method, Cheng et al. [57] used a transgene-free chemical-induced method, Ma et al. [58] used a combination of two lentiviruses, Drouin-Ouellet et al. [59] used a single-vector method, Kim et al. [60] used BAM reprogramming methods with additional factors, and Hu et al. [35] used a small molecule with a neural maturation media.

**Table 1:** Summary of studies included in the review

*Reference*	*iN characteristics*	*AD phenotype and biomarkers*	*Epigenetics*	*Electrophysiological*	*Genetics*	*Days in vitro*
*Herdy et al., 2022*	NeuN, β-III tubulin, Vimentin-positive.	Not reported.	Donor-specific iNs capture transcriptomic, mitochondrial, and epigenetic aging signatures in aging iNs.	Multi-electrode array.	Gene ontology analysis of upregulated genes identified a significant enrichment of genes related to extracellular matrix reorganization, including metallopeptidases, collagenases, and glypicans.	21
*Kim et al., 2022* [61]	β-III tubulin, NeuN, MAP-2, Synapsin- VGLUT1-positive.	In iNs, Aβ oligomers were identified at day 7 and increased until day 25. P-tau was significantly higher at day 7, compared to day 14. The number of Aβ_42_/TUJ1-postives iNs were significantly higher in *APOE* *ɛ*4 positives. *APOE* *ɛ*4 induction from the Aβ-seeding stage resulted in significant increase in the Aβ_42_/Aβ_40_ ratio in the AD patient iNs.	Not reported.	Not reported.	Gene set enrichment of AD patient iNs expressing *APOE* *ɛ*4 from the Aβ-seeding stage displayed a significant amount of DEGs (FC≥1.5), including 115 upregulated and 88 downregulated genes. The upregulated genes, including *IGFBP3*, *IGFBP5*, and *BMP2*, are particularly related to the insulin-like growth receptor signaling pathway.	25
*Traxler et al., 2022* [62]	β-III tubulin, NeuN, MAP-2, PKM2, PSD-95, Synapsin-positive.	Not reported.	Nuclear PKM2 activity alters the neuronal epigenetic landscape.	Electrophysiological analysis of iNs from AD and controls revealed mature physiological. properties and strong intrinsic excitability, and many iNs displayed voltage responses with characteristic features that indicated the action of specific voltage-activated membrane currents.	Gene co-expression modules overlapping between AD iNs and postmortem AD brains point toward aberrant metabolic regulation.	21
*Mertens et al., 2021* [64]	β-III tubulin, NeuN, MAP-2 -positive. iNs consisted primarily of glutamatergic (VGLUT1 positive) neurons and a minor fraction of GABA-positive neurons.	iNs from FAD patients showed increased Aβ42/Aβ40 ratios in conditioned media. Adult-like splicing patterns and MAPT protein variants in iNs. Capillary Western blot analysis for t-tau shows a wide distribution of tau species in iNs.	Epigenetic landscape profiling revealed an underlying aberrant neuronal state that shares similarities with malignant transformation and age-dependent epigenetic erosion.	Not reported.	778 highly significant differentially expressed genes were identified in iNs. Most genes were regulated in the same direction, indicating that viral treatment affected the fate of the cells into a common path. Gene set enrichment analysis of overlapping AD genes showed that they were enriched for mature neuronal categories such as synaptic transmission and oxidative phosphorylation.	21
*Cheng et al., 2021* [57]	β-III tubulin, NeuN, Tuj1.	Amyloid-β (N-terminal). iNs from Human fibroblasts expressing *APOE* *ɛ*3 or *APOE* *ɛ*4 display different contents of β-CTF/Aβ in endosomes and autophagosomes.	Not reported.	Not reported.	Not reported.	Not reported.
*Ma et al., 2020* [58]	β-III-tubulin, MAP-2, Synapsin 1, neurofilament 200, ChAT, Basal forebrain cholinergic neurons (BFCNs) neurotrophin receptor p75NTR positive cells.	iNs demonstrated time dependent tau hyperphosphorylation and dysfunctional nucleocytoplasmic transport.	Not reported.	Show electrophysiological properties of mature neurons.	qRT-PCR showed robust expression of genes enriched in neurons (*MAP-2, MAPT, CALB1) and BFCNs (ISL1, NKX2.1, CHAT, VACHT, ACHE, TRKA*), whereas the motor neuron-specific marker HB9 was not expressed.	28
*Hu et al., 2015* [35]	β-III-tubulin, MAP-2, VGLUT1, Tau, Synapsin 1.	APP mutation line: Increased extracellular Aβ_42_. Increased extracellular Aβ_42_/Aβ_40_. Increased p-tau and t-tau. *PSEN1* mutation lines: Variable Aβ production results. No change in p-tau or t-tau.	Not reported.	Active neuronal network formation. Electrophysiological response to exogenous L- glutamic acid and GABA.	Not reported.	12
*Kim et al., 2017* [60]	β-III-tubulin, MAP-2, VGLUT1-positive.	Accumulation of Aβ- polymers and increases in Aβ_42_-positive cells, and p-tau in SAD.	Not reported.	Action potentials and sodium and potassium currents.	Reported changes in gene expression related to *APP* processing (*BACE2, CLU, DSG2, PLAU, MME*) in *APOE* *ɛ*3/*ɛ*4 SAD line compared to *APOE* *ɛ*3/*ɛ*3 healthy control line.	40
*Drouin- Ouellet et al., 2017* [59]	β-III-tubulin, MAP-2, Synapsin1-positive.	Not reported.	Not reported.	Spontaneous postsynaptic activity was recorded.	qPCR analysis revealed a major increase in the neuronal genes (*NCAM, MAP2, MAPT, SYNAPSIN, SNCA, and SYNAPTOPHYSIN*) in every line converted, independently of the disease status of the donor.	30

**Table 2:** Summary of reprograming protocols

*Study id*	*ID/ vendor*	*Disease Duration (y)*	*Cells source*	*Age*	*Gender*	*AD type*
*Herdy et al., 2022* [63]	5835 5816 5784 5808 5836 5785 5837 5648 5620 5533 654 2800 2991 3053 3093 3113 3121 3131 3158 8020 8097 8149 8175 840 C HANS HR KP	unknown unknown unknown unknown unknown unknown unknown unknown unknown unknown unknown unknown unknown unknown unknown unknown unknown unknown unknown unknown unknown unknown unknown unknown unknown unknown unknown	Skin biopsy, antemortem Skin biopsy, antemortem Skin biopsy, antemortem Skin biopsy, antemortem Skin biopsy, antemortem Skin biopsy, antemortem Skin biopsy, antemortem Skin biopsy, antemortem Skin biopsy, antemortem Skin biopsy Skin biopsy Skin biopsy Skin biopsy Skin biopsy Skin biopsy Skin biopsy Skin biopsy Skin biopsy Skin biopsy Skin biopsy Skin biopsy Skin biopsy Skin biopsy Skin biopsy Skin biopsy Skin biopsy Skin biopsy	F F F M F F M M M M F M M M M M M F F F M F M M M N/A N/A	85 83 89 98 93 87 90 70 86 88 90 80 89 82 81 83 75 80 88 76 78 86 83 56 41 N/A 53	N/A N/A N/A N/A N/A N/A N/A N/A N/A N/A AD *APOE* *ɛ*3/*ɛ*4 AD *APOE* *ɛ*3/*ɛ*4 AD *APOE* *ɛ*3/*ɛ*3 AD *APOE* *ɛ*4/*ɛ*4 AD *APOE* *ɛ*3/*ɛ*3 AD *APOE* *ɛ*2/*ɛ*3 AD *APOE* *ɛ*3/*ɛ*3 AD *APOE* *ɛ*3/*ɛ*4 AD *APOE* *ɛ*3/*ɛ*3 AD *APOE* *ɛ*3/*ɛ*4 AD *APOE* *ɛ*3/*ɛ*3 AD *APOE* *ɛ*3/*ɛ*3 AD *APOE* *ɛ*3/*ɛ*3 AD *APOE* *ɛ*3/*ɛ*3 AD *APOE* *ɛ*3/*ɛ*3 AD *APOE* *ɛ*3/*ɛ*3 AD *APOE* *ɛ*3/*ɛ*3
*Kim et al., 2022* [61]	AG05810/Coriell AG04402/Coriell AG06848/Coriell AG09908/Coriell AG06869/Coriell	unknown unknown unknownunknown unknown	Skin biopsy, antemortem Skin biopsy, antemortem Skin biopsy, antemortem Skin biopsy, antemortem Skin biopsy, antemortem	79 47 56 81 60	F M F F F	LOAD *APOE* *ɛ*3/*ɛ*4 LOAD *APOE* *ɛ*3/*ɛ*4 FAD, type 2, *PSEN1* FAD, type 4, *PSEN2* SAD
*Traxler et al., 2022* [62]	8149 3093 2800 654 3053 3113 3131 8020 3121 HR 840 C	unknown unknown unknown unknown unknown unknown unknown unknown unknown unknown unknown	Skin biopsy Skin biopsy Skin biopsy Skin biopsy Skin biopsy Skin biopsy Skin biopsy Skin biopsy Skin biopsy Skin biopsy Skin biopsy	79 81 80 88 82 83 80 76 75 57 56	F M M F M M F F F M M	Classical AD, *APOE* *ɛ*3/*ɛ*3 Classical AD, *APOE* *ɛ*3/*ɛ*3 Non-classical AD, *APOE* *ɛ*3/*ɛ*4 Dementia Braak stage 3 tau, Aβ pathology, *APOE* *ɛ*3/*ɛ*4 Non-classical AD, *APOE* *ɛ*4/*ɛ*4 Non-classical AD, *APOE* *ɛ*3/*ɛ*2 Non-classical AD, *APOE* *ɛ*3/*ɛ*4 Classical AD, *APOE* *ɛ*3/*ɛ*4 Non-classical AD, *APOE* *ɛ*3/*ɛ*3 FAD, (APP-V717F), *APOE* *ɛ*3/*ɛ*3 FAD, (PS1-A246E), *APOE* *ɛ*3/*ɛ*3
*Mertens et al., 2021* [64]	8149 3093 2991 8097 2800 654 3053 3113 3131 8020 8175 3158 3121 KP HR 840C	unknown unknown unknown unknown unknown unknown unknown unknown unknown unknown unknown unknown unknown unknown unknown unknown	Skin biopsy Skin biopsy Skin biopsy Skin biopsy Skin biopsy Skin biopsy Skin biopsy Skin biopsy Skin biopsy Skin biopsy Skin biopsy Skin biopsy Skin biopsy Skin biopsy Skin biopsy Skin biopsy	79 81 89 78 80 88 82 83 80 76 83 75 75 53 57 56	F M M M M F M M F F M F F M M M	Classical AD, *APOE* *ɛ*3/*ɛ*3 Classical AD, *APOE* *ɛ*3/*ɛ*3 Non-classical AD, *APOE* *ɛ*3/*ɛ*3 Classical AD, *APOE* *ɛ*3/*ɛ*3 Non-classical AD, *APOE* *ɛ*3/*ɛ*4 Dementia Braak stage 3 tau, Aβ pathology, *APOE* *ɛ*3/*ɛ*4 Non-classical AD, *APOE* *ɛ*4/*ɛ*4 Non-classical AD, *APOE* *ɛ*3/*ɛ*2 Non-classical AD, *APOE* *ɛ*3/*ɛ*4 Classical AD, *APOE* *ɛ*3/*ɛ*4 Classical AD, *APOE* *ɛ*3/*ɛ*3 Classical AD, *APOE* *ɛ*3/*ɛ*3 Non-classical AD, *APOE* *ɛ*3/*ɛ*3 FAD, (*APP-SWE*), *APOE* *ɛ*3/*ɛ*3 FAD, (*APP-V717F*), *APOE* *ɛ*3/*ɛ*3 FAD, (*PS1-A246E*), *APOE* *ɛ*3/*ɛ*3
*Cheng et al., 2021* [57]	AG05810/Coriell AG10788/Coriell	unknown unknown	Skin biopsy, antemortem Skin biopsy, antemortem	79 87	F unknown	LOAD, *APOE* *ɛ*3/*ɛ*4 LOAD, *APOE* *ɛ*4/*ɛ*4
*Ma et al., 2020* [58]	AG06264/Coriell AG04402/Coriell AG21158/Coriell AG05810/Coriell	unknown unknown unknown unknown	Skin biopsy, antemortem Skin biopsy, antemortem Skin biopsy, antemortem Skin biopsy, antemortem	62 47 69 79	F M F F	LOAD *APOE* *ɛ*3/*ɛ*4 LOAD *APOE* *ɛ*3/*ɛ*4 LOAD *APOE* *ɛ*2/*ɛ*3 LOAD, *APOE* *ɛ*3/*ɛ*4
*Drouin-Ouellet et al., 2017* [59]	unknown	4	Skin biopsy	58	F	FAD (KM670/671NL)
*Kim et al., 2017* [60]	AG11414/Coriell AG05810/Coriell AG05770/Coriell AG06840/Coriell AG09908/Coriell	4 unknown 7.5 4 unknown	Skin biopsy, antemortem Skin biopsy, antemortem Skin biopsy, postmortem Skin biopsy, antemortem Skin biopsy, postmortem	3979705681	MFMMF	EOAD, described as SAD*, *APOE* *ɛ*3/*ɛ*4 SAD, *APOE* *ɛ*3/*ɛ*4 Unknown AD FAD, *PSEN1*, *APOE* *ɛ*3/*ɛ*3 FAD, *PSEN2*, *APOE* *ɛ*3/*ɛ*3
*Hu et al., 2015* [35]	FAD109/Xiangya Hospital FAD131/Xiangya Hospital FAD132/Xiangya Hospital FADA16/Xiangya Hospital	unknown unknown unknown unknown	Skin biopsy Skin biopsy Skin biopsy Skin biopsy	45 48 38 45	F F F F	FAD *APP*: p.V717I FAD *PSEN1*: p.I167del FAD *PSEN1*: p.A434T FAD *PSEN1*: p.S169del

Classical AD pathology (amyloid plaques Thal stage 5 and/or extensive neuritic plaques, and Braak stage 6 neurofibrillary tau pathology. Non-classical AD: these cases had neurodegenerative co-morbidity. Autopsies showed hippocampal atrophy with signs for hippocampal sclerosis and TDP43 inclusions, and only partially showed significant amyloid or tau pathology. **FTDP-17: Frontotemporal dementia and parkinsonism linked to chromosome 17; *Coriell vendor described case as early on FAD with dementia from age 35. Herdy et al. [63] reposted several sources of fibroblasts which includes: Coriell Institute Cell Repository, the University Hospital in Erlangen, and Shiley-Marcos Alzheimer’s Disease Research Center.

**Table 3:** Summary of reprograming protocols

*Study id*	*Induction methods*	*Differentiation media*	*Mature cell media*
*Herdy et al., 2022*	Lentivirus constructs EtO and XTP-Ngn2 : 2A:Ascl1 (E + N2A) or pLVXUbC-rtTA-Ngn2 : 2A:Ascl1	N2 supplement, B27 supplement, doxycycline, Laminin, dibutyryl-cyclic-AMP, human recombinant, Noggin LDN and A83-CHIR99021 Forskolin and SB.	N2, B27, GDNF, BDNF, dibutyryl, doxycycline, and laminin.
*Kim et al., 2022* [61]	Lentivirus constructs FUW-Ascl1, Brn2, Myt1l	N3 medium containing DMEM/F12, insulin, progesterone, transferrin, putrescine, laminin, FGF basic, BDNF, Forskolin, penicillin/streptomycin, and doxycycline.	N/A
*Traxler et al., 2022* [62]	Lentivirus constructs pLVXUbC-rtTA-Ngn2 : 2A:Ascl1	N2 supplement, B27 supplement, doxycycline Laminin, dibutyryl-cyclic-AMP, human recombinant Noggin LDN and A83-CHIR99021 Forskolin and SB.	N2, B27, GDNF, BDNF, dibutyryl, doxycycline, and laminin.
*Mertens et al., 2021* [64]	Lentivirus constructs pLVXUbC-rtTA-Ngn2 : 2A:Ascl1	N2 supplement, B27 supplement, doxycycline Laminin, dibutyryl-cyclic-AMP, human recombinant Noggin LDN and A83-CHIR99021 Forskolin and SB.	N2, B27, GDNF, BDNF, dibutyryl, doxycycline, and laminin.
*Ma et al., 2020* [58]	Lentivirus constructs Ascl1- IRES-GFP-T2A-Sox11 and LHX8-IRES-GBX1	FSK, LDN-193189, FGF2 DMEM/F12, N2, B27, penicillin/streptomycin.	C2 medium supplemented with FSK, BDNF, GDNF, and NT3 NGFβ.
*Drouin-Ouellet et al., 2017* [59]	Lentivirus constructs (Brn2, Ascl1, REST short hairpin RNA seq 1 &2).	Small molecules: CHIR99021 NDiff 227, LM-22A4, GDNF, NT3, db-cAMP, nCHIR99021, SB 431542, noggin, LDN-193189, valproic acid sodium salt.	Growth factors: (LM-22A4, LM-22A4, GDNF, NT3, db-cAMP.
*Kim, et al., 2017* [60]	Lentivirus constructs Transcription Factors: Brn2, Ascl1, Mytll, NeuroD1 + Plated on nanopatterned topography surface.	N3 medium, DMEM/F12, insulin, progesterone, transferrin, putrescine, laminin, bFGF, and penicillin/streptomycin.	Doxycycline administration (transgene activation) by 7 days.
*Hu et al., 2015* [35] & *Cheng et al., 2021* [57]	Chemical cocktail. Induction media: VPA, CHIR99021, Repsox, forskolin, SP600625, GO6983, Y-27632.	N/A	CHIR99021, forskolin, dorsomorphin, BDNF, GDNF, NT3 VCRFSGY (Valproic acid, CHIR99021, Repsox, Forskolin, SP600125, GO6983 and Y-27632).

#### Herdy et al., 2022 [63]

Herdy and colleagues followed a protocol developed by Mertens et al. [65] to robustly convert human fibroblasts. The study used material from AD brains, as well as iNs, and iPSCs. The study focused on the role of senescent cells in tissue dysfunction and AD.

#### Traxler et al., 2022 [62]

Traxler and colleagues followed a protocol developed by Mertens et al. [65] to robustly convert human fibroblasts. INs were derived from 11 individuals with AD and 11 age-matched, non-demented controls. Study focused on a new model for the metabolic changes that occur in neurons affected by AD. The study suggests that a Warburg-like metabolic transformation, in which neurons switch from oxidative phosphorylation to glycolysis, contributes to neuronal degeneration and cognitive decline in AD.

#### Mertens et al., 2021 [64]

Mertens and colleagues developed a protocol to robustly convert human fibroblasts. The material was derived from 16 AD patients (13 SAD and three FAD) and 19 age- and sex-matched, non-demented control donors.

#### Kim et al., 2022 [61]

This study investigated the impact of *APOE* *ɛ*4 on Aβ metabolism. The material was derived from five AD patient (two with autosomal dominant mutations and three without mutations). The authors focused on Aβ metabolism in AD iNs that transiently expressed *APOE* *ɛ*4.

#### Cheng et al., 2021 [57]

Cheng and colleagues studied two patients with LOAD (with *APOE* *ɛ*3/*ɛ*4 and *APOE* *ɛ*4/*ɛ*4 genotypes), and converted fibroblasts to iNs using a transgene free chemical-induced method [35], with the stated aim to investigate vitamin C-capability of APP degradation.

#### Ma et al., 2020 [58]

Ma and colleagues carried out conversion of fibroblasts from four LOAD patients using a novel combination of the two lentiviruses (ASLG). This study focused on iNs in co-culture with wild-type mouse astrocytes, to increase the longevity of cell lines. They included control iNs from four adult and two young non-AD humans.

#### Drouin-Ouellet et al., 2017 [59]

Drouin-Ouellet and colleagues focused not primarily on AD, but on the optimization of an induction method to efficiently reprogram dermal fibroblasts from elderly individuals using a single-vector system with a RE1-silencing TF (REST). The main objective of the study was to perform global gene expression analysis of fetal and adult fibroblasts to investigate the transcriptional response in the early stage of neural conversion and to better understand the reprogramming requirements specific to adult dermal fibroblasts. The study included cells from a range of familial and sporadic neurodegenerative disorders, including Parkinson’s disease, Huntington’s disease, and AD. Only minimal information was provided about the AD patient, other than it was FAD (*APP* mutation). No results were given on AD-specific phenotypes.

#### Kim et al., 2017 [60]

Kim and colleagues carried out conversion of fibroblast from two FAD patients, two SAD patients, and one unknown AD type patient, using BAM reprogramming methods with additional factors to produce neurons, and with tetO-mut *APP* to overexpress mutant *APP*.

#### Hu et al., 2015 [35]

Hu and colleagues developed a protocol to robustly convert human fibroblasts from four patients with FAD (one *APP*, and three *PSEN1* mutations) into functional iNs using a small molecule and a neural maturation media.

### AD related outcomes

The identified studies investigated various outcomes related to AD, including Aβ, *APP*, and tau. In the case of Drouin Ouellet et al. [59], Herdy et al. [63], and Traxler et al. [62], no results were given on AD-specific phenotypes of iNs, although the studies by Herdy et al. [63] and Traxler et al. [62] still present results that may inform on pathobiological mechanisms in AD, as summarized below.

#### Aβ and AβPP related outcomes

Hu et al. [35] showed that iNs derived from FAD patients with *APP* mutations had increased levels of extracellular Aβ_42_ and Aβ_42_/Aβ_40_ ratio compared to control cells. Mertens et al. [64] also reported that iNs from FAD patients (*n* = 2) carrying *APP* or *PSEN1* mutations had significantly higher levels of Aβ_42_/Aβ_40_ compared to iNs from age-matched controls (*p* < 0.0001), but the levels of Aβ_42_/Aβ_40_ in iNs from SAD patients (*n* = 9) were not significantly different from age-matched controls.

In their first study from 2017, Kim et al. [60] showed that iNs from a SAD patient with the *APOE* *ɛ*3/*ɛ*4 genotype exhibited accumulation of Aβ polymers, when compared to iNs from an *APOE* *ɛ*3/*ɛ*3 control. Both the control and SAD patient iNs had significant increases in Aβ_42_ positive cells after induction of AβPP-overexpression [60]. In their later study from 2022, Kim et al. [61] demonstrated that Aβ pathology was aggravated by the induction of *APOE* *ɛ*4 gene expression at an early Aβ “seeding stage” in AD iNs, where Aβ oligomers were identified as early as day seven, and levels increased until day 25. *APOE* *ɛ*4 induction in the Aβ “seeding-stage” resulted in a significant increase in the Aβ_42_/Aβ_40_ ratio in AD iNs. The number of Aβ_42_/Tuj1-positives iNs were significantly higher in *APOE* *ɛ*4 cells [61].

Cheng et al. [57] found that iNs from SAD patients with the *APOE* *ɛ*3/*ɛ*3 or *APOE* *ɛ*3/*ɛ*4 genotype had different contents and distribution patterns of β-CTF/Aβ (Aβ N-terminal-end specific monoclonal antibody) in endosomes and autophagosomes. *APOE* *ɛ*3/*ɛ*3 iNs had a uniform cytoplasmic accumulation of β-CTF/Aβ, and primarily in small cytoplasmic vesicles. In *APOE* *ɛ*3/*ɛ*4 iNs, β-CTF/Aβ appeared more scattered and clusters of β-CTF/Aβ were observed in the periphery of the cytoplasm and in the dendrites. After treatment with vitamin C, *APOE* *ɛ*3/*ɛ*4 iNs had changed distribution of β-CTF/Aβ, which became more concentrated to the centre of the cells and the clusters become less prominent, more similar to *APOE* *ɛ*3/*ɛ*3 iNs.

#### Tau related outcomes

In Ma et al. [58], iNs from SAD patients displayed tau hyperphosphorylation, was time-dependent and could be exacerbated by treatment with Aβ oligomers. No difference between patient and control iNs was observed at 28 days, but at 52 days post-infection the patient iNs had elevated p-tau in the somas when compared to the control. These phenotypic differences were observed later in the sample pairs from the younger age groups (phenotypic differences were observed at 62 days post-viral infection and become more apparent at 78 days) [58]. In Hu et al. [35], the levels of p-tau and t-tau were ∼2-fold increased in the iNs from the FAD patient with an *APP* mutation, but not in iNs from three FAD patients with *PSEN1* mutations, compared to controls [35]. In their 2017 study, Kim et al. [60] showed that (control) *APOE* *ɛ*3/*ɛ*3 and (SAD patient) *APOE* *ɛ*3/*ɛ*4 iNs had significant increases in tau phosphorylation after induction of AβPP overexpression. In their 2022 study, they showed that p-tau was increased following *APOE* *ɛ*4 induction at the Aβ seeding stage [61]. Mertens et al. reported (in supplementary data) that tau species were found in all iNs, but no comparison was provided between AD and control groups [64].

#### AD-dependent alterations in genetic and metabolic networks

Mertens et al. [64] focused on differential gene expression in AD iNs compared to control cells and found signs of upregulated markers of stress, cell cycle, and de-differentiation. Herdy et al. [63] found that AD brains had significantly higher proportions of neurons that expressed senescence markers, and that AD iNs exhibited strong transcriptomic, epigenetic, and molecular biomarker signatures, indicating a specific human neuronal senescence-like state. Traxler et al. [62] focused on a new model for the metabolic changes that occur in neurons affected by AD, and findings suggested presence of a Warburg-like metabolic transformation, in which neurons switched from oxidative phosphorylation to glycolysis, which may contribute to neuronal degeneration and cognitive decline in AD [63].

In their 2017 study, Kim et al. [60] analyzed the gene network derived from *APOE* *ɛ*3/*ɛ*4 iNs and revealed a strong interaction between *APOE* *ɛ*3/*ɛ*4 and another AD risk factor, the desmoglein 2 (*DSG2*) gene. Knockdown of the *DSG2* gene reduced the Aβ_42_ load in AβPP-overexpressing *APOE* *ɛ*3/*ɛ*4 iNs, demonstrating the functional importance of this interaction [60].

### Comparing reprogramming strategies

All identified studies demonstrated the potential of different reprogramming strategies to generate iNs for AD modelling. The studies highlight the importance of carefully selecting appropriate reprogramming factors and monitoring the stability of the induced neuronal phenotype.

#### Herdy et al., 2022 [63]

Herdy and colleagues used multiple lentiviruses. Human fibroblasts were infected with vectors containing EtO and XTP-Ngn2:2A:Ascl1 (*E* + N2A) or the combined Tet On system cassette consisting of the rtTAAdv driven by the UbC promoter, Ngn2:2A:Ascl1 under control of the TRE tight promoter, and a puromycin-resistance gene driven by the PGK promoter. and expanded in the presence of G418 and puromycin. The *UbC* gene is a regulator of ubiquitination, which regulates many cellular pathways, including in neuronal development, with synapse formation, synaptic pruning, and excitatory and inhibitory transmission in a brain [66]. The two pioneer TFs *Ascl1* and *Ngn2* both have a strong potential to induce neuron-like cells from fibroblasts [67].

#### Kim et al., 2022 [61]

Kim and colleagues used doxycycline-inducible tetO-FUW–based lentivirus contracts. FUW is a commonly used promoter that can drive high levels of transgene expression in a variety of cell types. When combined with the tetO promoter, the resulting tetO-FUW–based vector allows for tight regulation of transgene expression in response to tetracycline or its derivatives. The vector contained *Ascl1*, *Brn2*, and *Myt1l*., and were treated three times in two days. Then for *APOE* *ɛ*3 or *APOE* *ɛ*4 induction in human iNs were tread with doxycycline on day 7 or 14.

#### Traxler et al., 2022 [62]

Traxley and colleagues followed the Mertens et al. [64] protocol using the lentivirus contract pLVXUbC-rtTA-Ngn2:2A:Ascl1.

#### Mertens et al., 2021 [64]

Mertens and colleagues used a combination of TFs to induce neuronal differentiation, along with small molecules to enhance conversion. In sum, they used the lentivirus contract pLVX-UbC-rtTA-Ngn2:2A:Ascl1. This pLVX plasmid contains the UbC gene, a regulator of ubiquitination, which regulates many cellular pathways, including in neuronal development, with synapse formation, synaptic pruning, and excitatory and inhibitory transmission in a brain [66]. Reverse tetracycline-controlled transactivator (rtTA) is a part of Tet-Off and Tet-On expression systems, which is controlled by doxycycline [68]. They used activated cell sorting (FACS) to isolate and purify cells, achieving cultures of 90% β-III-tubulin-positive neuronal cells expressing NeuN.

#### Ma et al., 2020 [58]

Ma and colleagues used the double lentivirus contracts Ascl1-IRES-GFP-T2A-Sox11 and LHX8-IRES-Gbx1. The goal of this method was to derive induced basal forebrain cholinergic neurons. As the Mertens et al. [64] method, this also contains the Ascl1 gene, along with internal ribosome entry site (IRES), which is a translational enhancer. Some factors are primarily regarded as subtype-specifiers in this case, including Sox11 (for cholinergic neurons), which also has a considerable iN boosting efficiency, both for conversion of postnatal and adult skin fibroblasts [69]. The second half contains the Lhx8 gene (also known as *L3* and *Lhx*, *LIM homeobox*). Lhx8 plays a role in multiple functions, but in general acts as a cell fate mediator and is regulated by several TFs [70]. The *Gbx1* TF is essential for CNS development [71].

#### Kim et al., 2017 [60]

Kim and colleagues used a conversion strategy with BAM (*Ascl1, Brn2*, and *Myt1l*) + *NeuroD1*. Unlike the other BAM TFs, *Myt1l* is insufficient to induce iNs from fibroblasts on its own but is still considered an essential factor to induce iNs. *NeuroD1* is both complimentary and sufficient to induce key steps in neuronal differentiation [67]. The combination of BAM plus *NeuroD1* was used in the first direct conversion of neurons from human fibroblasts [72].

Kim and colleagues demonstrated that virus transduction can be complimented with a nanopattern topography. To overcome low efficiency, they applied modulating biophysical cues with surface nano-topography, produced from polyurethane acrylate with nanoscale grooved patterns with 300 nm height and groove width of 400 nm. The nanopattern acted as an efficient stimulant for direct lineage reprogramming human fibroblasts. Reprogramming for 15 days on nanopatterned substrates resulted in a 3-fold increase in the number of synapsin fluorescent protein-positive cells, relative to a control group [60].

#### Drouin-Ouellet et al., 2017 [59]

Drouin-Ouellet and colleagues implemented two TFs (*Brn2* and *Ascl1*) in combination with RE1-silencing complex (REST), a major neuronal gene repressor in non-neuronal cells, and the ageing-associated TFs and short hairpin RNA targeting REST (miRNA loops for miR-9/9 and miR-124) [67]. REST has neuron-inducing capabilities and can be regarded as ‘in-between’ pioneer and secondary factors. The REST complex acts as a potential barrier for reprogramming of mature human fibroblasts.

### Alternative to viral method: a chemical cocktail

Two papers used a chemical cocktail for induction [35, 57]. This chemical induction of neuronal cells from human fibroblasts might bypass a proliferative intermediate. The chemical cocktail had been optimized to a final combination of seven molecules, abbreviated VCRFSGY (Table 3). Treatment of human fibroblasts demonstrated a swift initiation of the cell cycle as early as day 3 *in vitro*. The combination works by the downregulation of fibroblast-specific genes and increased expression of endogenous neuronal TFs. The chemically induced neuronal cells resembled human iPSC-derived neurons with respect to morphology, gene expression profiles, and electrophysiological properties [35]. The main disadvantage of neuronal differentiation by chemical approach is a low efficiency [73], around 15% for human fibroblasts [35]. Another disadvantage is that the final product is a mixed population of neurons with different degrees of maturity and varying subtypes of neurons [73].

### Bias assessment

We considered two types of bias in this systematic review. The first aspect was the risk of bias of results within the included studies. Such bias could for example be present when there are unblinded outcome assessments. The studies included here did not mention any attempts to perform blinded analyses, which increases the risk for positive bias in the results. Another bias particular to iNs studies is the possibility of low conversion efficiency. The studies here reported conversion efficiencies ranging from 50% to 94%, but with different tools used to assess efficiency, which makes it difficult to strictly compare the studies (see details in the Discussion).

A second aspect of bias is the risk that there are missing studies, or missing results within studies (most typically negative results). Hypothetically, there may have been analyses done on iN cells from AD patients (also from other research groups than those included in this review) with non-significant results, which have not been submitted for publication. We note that there is a general lack of negative studies in this review, which suggest a risk for positive publication bias. There is also an overrepresentation of studies on FAD rather than SAD, and the studies that included iNs from SAD patients typically used cells with induced overexpression of *APP*.

We also note that although nine studies were identified, one research team contributed three of the studies, and another research team contributed two of the studies. The source material, the fibroblast cell lines, were overlapping between the studies within research teams, which increases the risk for bias for the overall results.

### Certainty assessment

With regards to certainty about results related to AD phenotypes, we note that most studies used different outcomes, although several studies measured AD biomarkers. Some of the studies found an increase in Aβ and p-and t-tau levels in FAD-induced neurons, although different FAD mutations appeared to affect Aβ and tau differently, and not all FAD mutations appeared to affect p-tau. Mutations in the *APP* gene were associated with increased Aβ levels. Few studies examined the effects of different *APOE* alleles on iNs, wherefore those results have lowercertainty.

## DISCUSSION

This systematic review of iN cells in AD identified nine papers which fulfilled our inclusion criteria. A wide variety of methods was used for cell reprogramming in these studies. Some of the studies explored AD-related phenotypes, such as differential levels of Aβ peptides and tau proteins. Most studies were very small, and most evidence related to AD phenotypes came from cells with autosomal dominant mutations, which are very rare in the general AD patient population. Taken together, these studies support the general concept of using iN cells as innovative cell models for AD research, but also show that more research is needed on larger cohorts, and on cells from patients with SAD, which is the most common form of the disease. Below we discuss specific aspects of the studies, regarding both the methods and challenges for cell conversion of fibroblasts from AD patients, and the AD phenotypes that have been exploredso far.

### Challenges in conversion of adult fibroblasts in AD

We note that there are both general challenges in converting somatic cells to iNs, and specific challenges related to the context of AD. Several protocols have been published and used (both in the papers included in this review, and in other non-AD iN papers), but the efficiency of the methods may be variable due to both known and unknown factors. For example, there is a striking range of the published efficacy of BAM TFs for neuronal reprogramming, ranging from 1.1% to 96% (reviewed in [74]). Several factors may contribute to low efficiency, including cell culture passage number (Drouin-Ouellet et al. [59] reported passages ranging from 3 to 10; Cheng et al. [57] reported cell passages between 6 and 15, and Herdy et al. [63] reported a passages rage 10–32; no info on passages was presented in the other papers), prolonged culturing of cells prior to conversion (no info was presented in the reviewed papers), and donor-specific factors. For example, younger donors’ cells are easier to reprogram than older donors’ [75]. This may have great importance for age-dependent diseases, such as AD. Different methodologies may have different susceptibility to such age-related reduced efficiency, with chemical treatments being more sensitive than vector-based conversion [76–78]. For the reviewed studies, this may be a problem for the chemical method used by [35].

As was pointed out in [60], the topography of the culture is a key factor for conversion efficiency. Enhanced topography resulted in a 3-fold increase of efficiency, and iNs on nanotopography had positive MAP2 and VGLUT1 15 days after reprogramming. The nanotopography of a surface plays a significant role in cell behavior [79]. Nanostructuring can be used to either promote or discourage cell adhesion by mimicking the extracellular matrix, and can affect both cell adhesion, migration, and spreading [80]. Surface topographies are even more influential than surface chemical cues in determining the alignment tendency of cells [81]. Nanostructuring may thereby constitute an intermediate step between standard 2D and more complex 3D models [82], which even more closely mimic *in vivo* CNS architecture.

Drouin-Ouellet and colleagues [59] reported neuronal reprogramming specifically adapted for the conversion of dermal fibroblasts of elderly donors. Such vectors have the potential to increase the yield by overcoming a hurdle in the way of using adult fibroblasts for iNs. They also show that the passage number of the starting fibroblast culture does not impact the reprogramming efficiency, at least up to 10 passages [59]. This is important as one biopsy could provide sufficient material for large-scale disease modelling, drug screening, and transplantation studies.

#### The role of different transcription factors

Different approaches have been implemented in the included papers. Seven papers used viral vectors and virus transduction [58–60, 62], but with different combinations of genes-conversion factors. Methods that rely on viral constructs strongly depend on conversion factors, which can be grouped into three categories: pioneer factors, secondary factors, and subtype factors. The majority of efficient iN protocols involve at least one “pioneer” factor (e.g., *Ngn2, Ascl1*, and *NeuroD1*) [67]. *Ngn2* alone can convert up to 90% of human fibroblasts into iNs, and Ascl1 alone can also induce neuron-like cells from fibroblasts. Secondary factors (e.g. *Mylt1, Brn2, Olig2*) do not induce conversion on their own and are instead used to achieve increased efficiencies and neuronal qualities [67]. Subtype factors (e.g., *Gata2, Lmx 1a/b, FoxA2*) can dictate subtype identity and are added to induce a desired neuronalsubtype [67].

Overall, in the nine different studies, five different conversion protocols were used, illustrating a lack of consensus on a preferred or optimal method for generating iNs. Direct reprogramming protocols continue to be developed and refined. The different protocols used in neural reprogramming include several different TFs, each with their own function that can drastically affect conversion rate as well as generated neuronal type. Studies in this systematic review had some overlapping pioneering TFs with different secondary factors. TFs play a decisive role in the subtypes of neurons and play a main role in efficiency conversion [75, 78]. TFs that are used for direct reprogramming to produce iNs are typically on-target factors, like *Ascl1, Ngn2*, and *NeuroD1*. They act by binding to specific chromatin regions to induce trans-differentiation and remain at the region until switched off in doxycycline-inducible systems [67]. Different TFs are required to directly reprogram cells into specific iNs subtypes, since the efficiency of different on-target pioneer TFs differ by cell types [83]. *Ascl1* and *Ngn2* play key role in mammalian brain development, during which they instruct stem and progenitor cells in diverse brain regions toward different neuronal type such as GABAergic and glutamatergic neurons [84]. Furthermore, Ascl1 can induce conversion of fibroblasts, but not keratinocytes [83]. This is different from off-target iPSC reprogramming TFs that can equally reprogram a broad variety of somatic cells into pluripotency. The difference is caused by on-target pioneer TFs requiring a more specific epigenetic signature and pre-existing histone modifications in order to bind to closed chromatin [67]. Additional conversion factors may primarily impact on the neuronal subtype generated, and future studies will be required to determine the optimum combination depending on the research goals.

At the moment, the TF-mediated approach might represent the most feasible path to produce mature, functional cell identity. All studies with vector approaches relied on Ascl1, which is essential to obtain neurons from human fibroblasts. As mentioned before, *Ascl1* alone or together with *Myt1l* and *Brn2* (used in Kim et al. [61] and Drouin-Ouellet et al. [59] papers, respectively, as second TFs) is able to generate glutamatergic iNs [85]. Ma et al. [58] opted for *Sox11*, another essential regulator of neuronal fate and survival. An extensive comparison of TFs between studies is difficult, and deeper systems-level analyses may be needed to find the optimum vector complex to manipulate adult human fibroblast with the highest efficiency. It is very rare with published systematic comparisons between different conversion methods in the same lab. We note that it may also be important to consider the reprogramming roles of additional regulators of cell fate such as miRNA loops for miR-9/9 and miR-124. The addition of such regulators seems to be beneficial in cases where conversion efficiency and maturation of iNs are promoted by the introduction *Brn2, Ascl1, Myt1 L*, and *NeuroD2* [86].

#### Small molecules

A vector-based conversion can be further supplemented with small molecules (Table 3). Small molecules are nongenetic boosters of iNs, that can block or activate signaling pathways involved in direct conversion or that are known to benefit neuronal differentiation, maturation, or survival. They are used to increase efficiencies, and to obtain iNs with better neuronal qualities. Furthermore, multiple studies have shown that small molecules can convert human astrocytes or fibroblasts alone, without genetic vectors, into functional neurons [77, 87]. Different differentiation and maturation media were used in each study in this review, but since the studies also differed in TFs, it is difficult to establish the importance of specific choices of small molecules.

#### Conversion efficiency

We note that the studies in this review used different approaches to determine conversion efficiencies. Mertens et al. [64] reported efficiency rates over 50% (DAPI and positive for expressing β-III-tubulin) independent of donor age. Drouin-Ouellet et al. [59] reported efficiency rates 92–94%, where the percentage of TUJ1 and GFP positive cells among total GFP positive cells was used to calculate conversion efficiency. Kim et al. [60] reported around 65% VGLUT1/MAP-2 positive cells on day 8. Ma et al. [58] reported conversion efficiency at around 92–94%, which was calculated as the percentage of TUJ1 and GFP positive cells among total GFP positive cells at 28 days. Cheng et al. [57], Herdy et al. [63], Kim et al. [61], and Traxley et al. [67] did not report efficiency rates. However, Herdy et al. [63] used FACS-based purification, to obtain mature iNs. The lack of consensus on how to establish conversion efficiency makes it difficult to directly compare the studies even with reportedefficiencies.

### Epigenetic component

As outlined above, one major hypothetical advantage of iN cells compared to iPSCs in diseases where advanced age is a primary risk factor, such as AD, is that iNs retain epigenetic age-related signatures.

Specific epigenetic signatures of cells also play an important role in direct reprogramming. Although there is no consensus regarding epigenetic involvement in particular genes, there is evidence supporting overall epigenetic connections with AD mechanisms [88]. Epigenetic mechanisms, particularly those of DNA methylation and histone acetylation and deacetylation, show dysregulation in AD when compared to healthy patients [89, 90]. Among the epigenetic mechanisms, histone modifications are relatively more established in AD, and treatments that are based on histone deacetylase inhibition have shown promising potential in drug development [52], which even further underscores the importance of epigenetic retention in AD models. Three identified studies, all from the same team of researchers (Mertens et al., [64], Herdy et al. [63], and Traxley et al. [67]), reported on epigenetics. Epigenetic landscape profiling in these studies demonstrated that AD iNs have an atypical neuronal state that shares similarities with malignant transformation and age-dependent epigenetic erosion [62]. Future studies can in more detail clarify to what degree fibroblast-derived iNs reflect old adult brain stages.

### AD-related features in iN cells

Six of the nine studies (all except Drouin-Ouellet et al. [59], Herdy et al. [63], and Traxler et al. [62]) attempted to show effects on specific AD-related features in the iN cells, mainly related to *APP*, Aβ, or tau. In living humans, biomarkers related to Aβ, and tau measured with positron emission tomography or in cerebrospinal fluid (CSF) or plasma have been used for AD diagnosis and to study disease mechanisms. For example, CSF Aβ_42_ [91] and plasma Aβ_42_ [92] are reduced in the presence of Aβ pathology in the brain. In patients with FAD, CSF Aβ_42_ may be elevated [93]. It is therefore natural to study alterations in Aβ and tau in iN cells.

Four studies [60–62] measured AD-related biomarkers, such as Aβ and p-tau (Table 1). Mertens et al. [64] demonstrated that iNs from FAD (but not SAD) patients developed an increased extracellular Aβ_42_/Aβ_40_ ratio. In their 2022 study, Kim et al. [61] demonstrated that iNs with *APOE* *ɛ*4 have increased p-tau accumulation in cell bodies and dendrites at the Aβ-seeding stage, but *APOE* *ɛ*4 induction in healthy iNs did not affect p-tau accumulation. Meanwhile, thioflavin T-positive deposits in AD iNs were significantly increased by *APOE* *ɛ*4 induction at the Aβ seeding stage. The authors suggested that the induction of *APOE* *ɛ*4 at the early Aβ seeding stage accelerated both p-tau and Aβ aggregation, but induction of *APOE* *ɛ*4 later, at the “aggregation stage” of Aβ, did not have those effects. In Kim et al. [60] when cultured on nano-topography, both SAD iNs with or without overexpression of *APP* exhibited accumulation of Aβ. Additionally, Kim et al. [61] reported a significant increase in Aβ_42_-positive cells and p-tau in *APP*-overexpressing iNs with *APOE* *ɛ*3/*ɛ*3 or *APOE* *ɛ*3/*ɛ*4, as early as ten days after induction, indicating the potential usefulness of nanopattern for iN AD models. Hu et al. [35] reported increased extracellular Aβ_42_, Aβ_42_/Aβ_40_ ratio, p-tau and t-tau in FAD iNs compared to controls.

One study [57] demonstrated that iNs with genotype *APOE* *ɛ*3 and *APOE* *ɛ*4 had different contents of *APP* degradation products (β-CTF/Aβ) in endosomes and autophagosomes. β-CTF/Aβ in *APOE* *ɛ*4 iNs appeared more scattered present in the periphery of the cytoplasm, in the dendrites, and sometimes at the cell surface. Meanwhile, iNs with *APOE* *ɛ*3 displayed uniform distribution in the cytoplasm, primarily located near small cytoplasmic vesicles. Furthermore, no clustered accumulation was observed.

Ma et al. [58] demonstrated that SAD iNs had time-dependent tau hyperphosphorylation and dysfunctional nucleocytoplasmic transport. As no significant differences were observed between control and AD at the early stage, 28 days post-infection (dpi), was observed using western blotting. However, immunohistochemistry analysis showed much elevated p-tau in the somas, at 52 dpi when compared AD (62 years, *APOE* *ɛ*3/*ɛ*4) to the control (70 years APOE *ɛ*3/*ɛ*3). Furthermore, the p-tau difference was delayed compared to younger controls (47 years, APOE *ɛ*3/*ɛ*3) and AD patients (47 years, APOE *ɛ*3/*ɛ*4). The increased p-tau phenotype in AD iNs was not observed at the early time point 52 dpi, but it was evident at 62 dpi and became even more significant at 78 dpi. Thus, Ma and colleagues [58] concluded that longer term cultures could be vital when investigating biomarkers.

Kim et al. [60] studied effects of the AD risk gene APOE on cell survival. They observed no overall difference between the number of iNs with APOE *ɛ*3/*ɛ*3 or APOE *ɛ*3/*ɛ*4 (without *APP* overexpression), suggesting no evidence of APOE *ɛ*4-associated neuronal loss. However, SAD APOE *ɛ*3/*ɛ*4 iNs with additional overexpression of the *APP* gene had a noticeable decrease in the number of cells positive for β-III tubulin and MAP2, potentially suggesting decreased cell survival. In the Kim et al. study [60], morphological changes and a reduced number of iNs with overexpression of *APP* suggests that increased synthesis of *APP* itself may be responsible for such morphological changes, which is also seen in studies using SH-SY5Y cells. These changes are most likely due to increased susceptibility to mitochondrial oxidative stress and activation of the intrinsic apoptotic cascades [94]. Directed overexpression of single genes provides valuable information and could be used for drug screening, as it seems iNs without overexpression would require a longer time to express AD phenotype. Aβ accumulation in mitochondria, endosomes, and autophagosomes is age-related [95]. Therefore, the differences in contents of β-CTF/Aβ in endosomes and autophagosomes in iNs with APOE *ɛ*3 or APOE *ɛ*4 may also be an early morphologic abnormality.

Mertens et al. [64], Herdy et al. [63], Traxler et al. [62], and Kim et al. [60] both studied alterations in gene networks in AD iNs. Mertens et al. [64] found evidence of a generally altered genetic environment, described as a “hypo-mature neuronal identity”. Herdy et al. [63] found evidence of cellular senescence, and Traxler et al. [62] found evidence of an altered metabolic state in AD iNs. Kim et al. [60] found evidence implicating a role for a specific gene, *DSG2*, which was suggested to also be relevant for Aβ accumulation. Together, these studies support the use of iNs to study alterations in global genetic environments in AD.

### Limitations

This systematic review is limited by the low number of studies eligible for review. Given the low number of studies, and the varying methods and outcomes among the studies, it was not possible to perform a quantitative meta-analysis of AD-related outcomes. However, there are several limitations to the current studies that need to be addressed. Most studies are small, with an overrepresentation of studies focusing on FAD, and a lack of studies on SAD. Most human AD patients have the SAD variant, without genetic mutations, which may limit the generalizability of FAS-centered studies. However, within AD research in general, FAD studies have been very informative also for AD in general, for example with studies of biomarker trajectories and early disease mechanisms [96, 97]. There is a lack of standardization in methods for iNs generation and differentiation, and some studies lack details on conversion rates, cell passage numbers, and formal statistical tests for key outcomes. Different protocols may result in variable levels of maturation and different AD-related biomarkers, making it difficult to compare results across studies. Standardization of protocols could help address this issue and ensure reproducibility of results. Long-term 3D cultures might enhance our understanding of long-term effects in AD [98, 99].

### Conclusion

Despite considerable interest in iN cells as models for neurodegenerative diseases [100], there are still only few studies that present direct conversion of fibroblasts to iNs in human AD patients, and most of these studies are small. The studies provide promising results regarding the effects of different conversion systems and suggest that patient-derived iNs represent a valid model for studying AD pathogenesis. The iN cells could be a strategy to identify targets for new therapies, and perhaps even to test candidate therapeutics if robust AD-related cellular outcomes can be identified. Most iN findings related to AD-phenotypes came from FAD iNs. It should be a priority to extend these studies to larger SAD cohorts, for models that are broadly generalizable to the AD community.

## Supplementary Material

Supplementary Material

## Data Availability

The data used in this systematic review is available from the corresponding author upon reasonable request.
